# Studying the impoverishing effects of procuring medicines: a national study

**DOI:** 10.1186/s12914-019-0210-x

**Published:** 2019-07-31

**Authors:** Mohammadreza Amiresmaili, Zahra Emrani

**Affiliations:** 1 Department of Health Management, Economics and Policy Making, School of Management and Medical Informatics, Kerman University of Medical Sciences, Kerman, Iran; 2 Department of Health Management and Economics, School of Public Health, Tehran University of Medical Sciences, Tehran, Iran; 3 Health Management Research Center, Institute for Futures Studies in Health, Kerman University of Medical Sciences, Kerman, Iran

**Keywords:** Drug expenditure, Health equity, Iran, Impoverishment

## Abstract

**Background:**

One of the main treatment procedures is through medicine prescription. Considering the rising burden of drug costs, we conducted this study to estimate the impoverishing effects of medicine on Iranian households.

**Method:**

We carried out calculations based on the Iranian National Household Survey for the year 2013. Amoxicillin, atorvastatin and metformin were the drugs selected. Three different poverty lines were applied. Impoverishment was estimated for various scenarios. Additionally, the associations of some demographic factors were tested. Excel 2013 and SPSS v.19 were used.

**Results:**

Many households fell under the poverty line after purchasing drugs. Procuring original brand (OB) drugs caused more poverty than lowest-priced generic (LPG) equivalents. The logistic regression testing showed that the age, gender and literacy of the head of household and the size of the household were associated with impoverishment.

**Conclusion:**

This study showed that purchasing medicines increases the impoverishment risk of households. This risk is an index used to assess financial protection against health costs, which is in turn an indicator of health equity. The results will be of practical use for policymakers when addressing different scenarios of setting medicines prices as well as when considering alternatives for cost shifting for cross subsidies in pharmaceutical procurement.

## Introduction

One of the goals of the healthcare system is to make healthcare costs fair for people. In recent decades, increased costs of healthcare, caused by technology advances on the one hand and people’s increased expectations and knowledge on the other, have created some problems for financing healthcare [1]. In response to such issues the United Nations provided a set of policy actions, to address the challenge of financing and to achieve the sustainable development goals, which lead 193 United Nations member states to publish a post-2015 framework (Addis Ababa Action Agenda) to be implemented by members. Providing essential public services for all, improving national health system and trying to end poverty, hunger and malnutrition were emphasized [2, 3]. To this end, governments around the world have to take measures to identify the causes of poverty and to protect their people against poverty, impoverishment and hunger [3, 4].

Paying for health services or family’s health care cost is one of sources which might push families under poverty line [5]. A family’s healthcare cost is defined as the family’s entire economic contribution to the healthcare system, which is divided into two categories: out-of-pocket (OOP) payment and pre-payment. The main difference between the two concerns the pooling of risk across the entire population [6].

Studies have shown that, in countries with pre-payment systems, financial protection is better and healthcare costs lower [7, 8]. Providing financial justice for all in the health system is very important, albeit difficult, and leads to improved health outcomes, equity and financial protection, and consumer satisfaction. The number of households faced with catastrophic healthcare costs is a measure of fairness in the health economy [9]. However, this index is somewhat hidden from society, while falling below the poverty line is very prominent in society. Thus, an alternative is to look at “impoverishment”, which indicates the number of people that have fallen below the poverty line because of healthcare costs [10].

In Iran, a few studies have been conducted to estimate the healthcare costs that cause people to fall under the poverty line. Yazdi-Feizabadi and Akbari conducted a study on the effects of health expenditures on households’ impoverishment in Iran. They concluded that the mean proportion of households falling under the poverty line was 7.5% over the years 2008–2014 [11]. Rezapour et al. (2013) also studied poverty, in Tehran and based on 2013 data. Their study indicated that the poverty rate among households including healthcare costs was 4.38%, and that excluding healthcare costs was 3.6% [12].

Medication costs represent the highest category of healthcare costs for households [13]. The use of medication is very high among Iranian households and many factors have been identified explaining this excessive use of medication. Almost 53% of Iranians self-medicate, which is several times the world’s rate [14]. Additionally, changes of health status indicate changes in disease patterns from contagious to non-contagious and chronic diseases [15], and an increasingly aging population in the future [16]. Chronic diseases cause pain and disability, reduce quality of life and increase the need for medication [14]. The need for repeated doctors’ visits, high costs of visits, easy access to medication and illness expansiveness are among the factors that cause people to buy medications without a prescription [14, 17–19]. In such circumstances, all of the drug costs fall on the people. The continuation of this situation can cause financial problems and even treatment avoidance. Despite the importance of drug costs in Iran, so far, no study has been conducted to specifically investigate the impact of these costs on households’ financial indices. Therefore, the present study aimed to investigate the effect of medication costs on households and their impoverishment.

### Health system in Iran

In Iran the Ministry of Health and Medical Education (MOHME) is not only responsible for education and training but also provides health care services [20]. This organization with the help of universities of medical sciences in different provinces oversees and monitors the operation of health system [21]. The Iranian health system is an insurance based system [22]. There are both governmental and largely employer-based independent insurance organizations in health care financing system [23, 24]. The governmental insurance include: A) Iran’s Health Insurance Organization (IHIO) which consists of five funds (civil servants fund, Iranian medical insurance fund, the rural and nomads fund, the universal health insurance fund, other social strata fund) [25], B) the Social Security Organization (SSO) which covers people subject to labor law, C) the Armed Forces Medical Service Organization (AFMSO): for members of the military and their dependents, D) Imam Khomeini Relief Foundation (IKRF) which is designed for covering uninsured poor. And finally there are some independent organizations which insure their own employees including Iranian national oil company, Islamic Republic of Iran Broad casting (IRIB), banks [23, 24]. Insurers receive some governmental resources (mostly from revenue of natural resources sale, especially oil) in addition to the premium and sometimes receive donations [21]. There are also Private Health Insurances which are funded through premium [25]. Existence of different insurances (with different benefit packages) has caused different coverage conditions. While a fair financing system has been confirmed in the mission of the Iranian health system [26]. So the implementation of health reform plans, redefinition of procedures and changes in financing system structure are to reach this mission.

### Research method

This cross-sectional study was conducted using the data sets of the Iranian National Household Survey, conducted by the Statistical Center of Iran, regarding family expenses and incomes related to 2013. The study population consisted of all urban and rural households in Iran. Sampling was done by the Statistical Center of Iran through stratified multistage sampling. It has three steps. First categorizing and head counting regions. Second, urban blocks and rural hamlets are selected from the regions. In the third step households are selected. The sample size is optimized according to the aim of this plan to estimate average income and expenditure of a household. To achieve better estimations, recruiting of sample households were distributed during all months of the year [27]. The total number of sample households was 38,244, of which 18,854 households were living in the city and 19,390 in rural areas.

### Data collection

Studies that measure the ability to pay for a service or product require three types of information: (a) the price of the product or service, (b) the household income or household consumption expenditure and (c) a threshold [28].

- In this study, to examine the payment ability of the sample households in regards medication costs, three types of medications, Amoxicillin Cap, Metformin Tab, and Atorvastatin Tab, were selected. These medications have been identified as popular and widely used by the Food and Drug Administration [29], and their retail price was set as the base line.
- Selecting “income” means the household does not have any other resources (for example savings or borrow) to pay for health. In this study, we preferred to use the “household consumption expenditure” data, based on the data set related to the year 2013.
- Three types of poverty line were taken into account as threshold.

### Data analysis

To calculate the households that had fallen below the poverty line due to the costs of medication, the direct OOP payments of each household to purchase medication (in addition to their health costs) were taken away from the total expenses of the household. That number was then compared with the household’s survival expenditure (as the threshold). If the figure was less than the survival expenditure, it indicated that the household had fallen under the poverty line. (The survival expenditure of a household is the minimum amount that the household must spend to survive in the community. The poverty line in this study was considered to be the household’s minimum survival expenditure). Particular attention must be paid in the calculation and that is to make sure the household has not been below the poverty line before spending their money on medication [18]. In other words in prospective method of impoverishment, first the household’s impoverishment because of their health cost (OOP) is calculated. Second the household’s impoverishment after paying for medication (in addition to their previous health cost) is calculated. Then these two figures are subtracted to obtain the real impoverishment after purchasing the medication.

There are different ways to determine poverty. Three types of poverty line were taken into account to obtain the results:

1. Since the poverty line was not announced by officials in 2013, the poverty line stated by economic experts was used ($140 per month for urban households and $84.5 per month for rural households), labelled the “informal poverty line” in this study [30].
2. Urban and rural poverty lines for 2013 were calculated as follows:
  Households were set ascending based on “food exp” (which is defined as food expenditure share of total household expenditure) data. Households were classified into 100 percentiles based on the “food exp” data. The 45th and 55th percentiles were identified. The average weight of equivalent food costs (which is food expenditure divided by equivalent household size) of those households situated between the 45th and 55th percentiles was calculated. As a result, the per capita survival expenditure, or poverty line, was achieved. The following formulas were used [31]:

$$ PL= food45\prec food{\exp}_h\prec food55 $$

$$ PL=\frac{\sum Wh\ast eqfood}{\sum Wh} $$

“Food exp” denotes the food expenditure share of total household expenditure:

$$ food{\exp}_h={food}_h/{\exp}_h $$

“Eqfood” refers to the food expenditure ratio of equivalent household size:

$$ {Eqfood}_h={food}_h/{Eqsize}_h $$

Equivalent household size (Eqsize) is:

$$ Eqsize={hsize}^{0.56} $$

3. The World Bank has determined a poverty line and prepared a basis for comparison between different countries. Therefore, the international poverty line was also considered in this study. The international poverty line is US $1.90 and $3.10 per day in 2011 purchasing power parity (PPP), which is convertible to each country’s domestic currency based on its PPP conversion factors. To convert international dollars into the local currency (Iranian Rial), the US dollar figure was multiplied by the PPP conversion factor (which is 5001/363). In the second step the resulted figure was multiplied by proportion of Consumer Price Index (CPI) of the year 2013 divided to CPI of 2011. In This way inflation between 2011 and the year in which the household surveys were conducted (2013) was adjusted for [32].

In this study, all variables were gathered on a monthly basis. Calculations were based on the Iranian Rial. Results are reported based on the US dollar. Excel 2013 and SPSS v.19 were used for the calculations.

### Scenarios

Ten scenarios of taking medicine were created. In each scenario, we calculated the percentage of households that fell below the poverty line due to medication costs. Since this study aims to obtain the results with prospective method of impoverishment calculations, in every scenario, the possibility of disease in the household was considered in the calculations. Amoxicillin (500 mg, 3 times a day, $0.11 per day), Atorvastatin (10 mg, once a day, $0.02 per day), and Metformin (500 mg, 3 times a day, $0.03 per day), used to treat sinusitis (53% prevalence) [33], hypercholesterolemia (41.6% prevalence) [34], and type 2 diabetes (8.5% prevalence) [35] respectively, were selected to create the scenarios. Lipitor ($0.39 daily) and Glucophage ($0.15 daily), original brand (OB) drugs used to treat hypercholesterolemia and diabetes respectively, were included in three of the scenarios.

## Results

In this section, the results of the analysis of households’ medication expenditure under the different scenarios are presented. Table 1 is provided in three parts. In the first part different poverty lines are inserted in the first column. Percentage of people under the poverty line before purchasing drug can be found in the second column. Other 10 columns show percentage of households below the poverty line after procurement of the drugs. Second part of the table shows percentage of households drawn under the poverty line because of purchasing drugs. Third part shows number of households which have been impoverished by expenditures on drugs.

**Table 1:** Impoverishment and unaffordability estimates relating to purchase of medicine under different scenarios

Impoverishment calculation scenarios		Before purchasing drug	Amoxicillin	Atorvastatin	Metformin	Amoxicillin & Atorvastatin	Amoxicillin & Metformin	Metformin & Atorvastatin	Amoxicillin & Metformin & Atorvastatin	Lipitor	Glucophage	Glucophage & Lipitor
Poverty lines												
Informal PL (per month)	Urban ($140)	27.6	27.8	27.7	27.8	27.9	28	28	28.2	30.4	28.6	31.6
	Rural ($84.4)	20.8	21	21	21.1	21.2	21.3	21.2	21.5	24.6	22.2	26
Calculated PL (per month)	Total ($74.7)	9.7	9.9	9.9	10	10.1	10.1	10.1	10.3	12.3	10.6	13.2
	Urban ($82.3)	5.8	5.9	5.9	6	6	6.1	6.1	6.2	7.5	6.4	8.3
	Rural ($68.7)	11.9	12	12	12.1	12.2	12.3	12.3	12.5	15.4	13.1	16.7
International PL ($3.10 PPP)	Total	0.2	0.3	0.2	0.3	0.3	0.3	0.3	0.3	0.7	0.4	1
International PL ($1.90 PPP)	Total	0	0	0	0	0.1	0.1	0.1	0.1	0.2	0.1	0.3
Absolute impoverishment and unaffordability of medicine under different scenarios (percentage)												
Informal PL	Urban households	–	0.2	0.1	0.2	0.3	0.4	0.4	0.6	2.8	1	4
	Rural households	–	0.2	0.2	0.3	0.4	0.5	0.4	0.7	3.8	1.4	5.2
Calculated PL	Total households	–	0.2	0.2	0.3	0.4	0.4	0.4	0.6	2.6	0.9	3.5
	Urban households	–	0.1	0.1	0.2	0.2	0.3	0.3	0.4	1.7	0.6	2.5
	Rural households	–	0.1	0.1	0.2	0.3	0.4	0.4	0.6	3.5	1.2	4.8
International PL ($3.10 PPP)	Total households	–	0.1	0	0.1	0.1	0.1	0.1	0.1	0.5	0.2	0.8
International PL ($1.90 PPP)	Total households	–	0	0	0	0.1	0.1	0.1	0.1	0.2	0.1	0.3
Absolute impoverishment and unaffordability of medicine under different scenarios (number of households)												
Informal PL	Urban households	–	20	8	3	–	–	–	–	220	16	–
	Rural households	–	21	16	5	–	–	–	–	307	23	–
Calculated PL	Total households	–	41	32	10	–	–	–	–	414	29	–
	Urban households	–	10	8	3	–	–	–	–	133	10	–
	Rural households	–	10	8	3	–	–	–	–	282	20	–
International PL ($3.10 PPP)	Total households	–	20	0	3	–	–	–	–	80	7	–
International PL ($1.90 PPP)	Total households	–	0	0	0	–	–	–	–	32	3	–

Table 1 shows that the number of households that fell below the poverty line due to drug costs was greater among rural than urban households. Various poverty lines have been used in the table. According to the poverty line we calculated for this study, 41 households would not have been able to pay for Amoxicillin. According to the international poverty line of $3.10 PPP, paying for Amoxicillin would have caused 20 households to fall below the poverty line. Based on the poverty line of $1.90 PPP, this drug would have been affordable for almost all households. Obviously, a higher poverty line causes unaffordability of drug costs in a greater number of households.

Furthermore, the proportion of families falling below the poverty line due to paying for Atorvastatin is significantly different to that falling below it due to purchasing Lipitor. The same is true for the use of Glucophage compared to Metformin. Therefore, it can be concluded that people’s ability to pay for OB drugs is much lower.

In this study, the risk of disease occurring in the society as a whole was assumed equal to the risk of disease occurring in the sample households [36]. In the scenarios where more than one drug was used in a household, the probability of more than one disease occurring in a household was calculated by multiplying together the prevalences of the diseases, regardless of the number of family members. This resulted in a smaller number than the probability of occurrence of only one disease. However, these probabilities do not seem logical. Therefore, the absolute impoverishment numbers in scenarios with more than one drug were not calculated, thus some of the spaces in Table 1 are empty.

Table 2 shows the association between impoverishment (using the poverty line we calculated, of $74.7 per month) and some household characteristics, based on the scenario in which Metformin and Atorvastatin were purchased.

**Table 2:** Regression results for determinants of impoverishment

Variables		Univariate Regression results			Multivariate Regression results		
		B	OR (CI 0.95%)	Sig	B	OR (CI 0.95%)	Sig
Gender of head of household	Male	–	1		–	1	
	female	1.239	3.451 (3.191–3.732)	0.000	0.842	2.321 (2.123–2.539)	0.000
Literacy of head of household	Literate	–	1		–	1	
	Illiterate	1.400	4.055 (3.788–4.342)	0.000	1.441	4.244 (3.845–4.639)	0.000
Size of household	Less than 5	–	1		–	1	
	Equal or more than 5	−0.119	0.887 (0.820–0.960)	0.003	0.197	1.218 (1.117–1.328)	0.000
Age of head of household	Less than 40	–	1		–	1	
	40 < age < 60	−0.486	0.615 (0.565–0.670)	0.000	−1.64	0.345 (0.313–0.380)	0.000
	60 < age < 80	0.500	1.649 (1.516–1.794)	0.000	−0.720	0.487 (0.436–0.544)	0.000
	More than 80	1.383	3.987 (3.484–4.562)	0.000	0.59	1.061 (0.906–1.243)	0.462

The relationships between demographic factors and households’ impoverishment were found to be significant in one-variable logistic regression tests. Female-headed households had 3.4 times greater odds of falling below the poverty than male-headed households. Illiterate-headed households had four times the odds of falling below the poverty line of literate-headed households. The probability of falling into poverty was less in households with more than five members. The likelihood of falling into poverty was less in households whose heads were between 40 and 60 years old than in those whose heads were either younger or older.

In multi-variable logistic regression test, all factors, except for having an elderly head, over 80 years old, were significant. Female-headed households, illiterate-headed households and having fewer than five household members were all among the risk factors affecting households’ poverty.

## Discussion

In Iran, due to the social and economic changes over the past 30 to 40 years, improvements in technology, and increased access to health services, some changes have taken place in the age structure of the population, the incidence of disease, and the causes of mortality. The aging population shows an increased risk of non-contagious diseases, besides the dispersion and potential risk of contagious diseases [15]. In this situation, healthcare should be able to cover the needs of the society more accurately and quickly [37]. Medication is an essential and effective part of healthcare that accounts for a major share of households’ expenditure [38]. Supply of and access to the highest variety of effective, healthy, high-quality and reasonably priced medication is one of the goals in Iran’s 20-year vision plan [39]. This study has shown that 0.2% of Iranian households cannot pay for Amoxicillin, a widely used medication. In addition, 0.2 and 0.3% of households are not able to pay for Atorvastatin and Metformin, respectively (based on our calculation of the poverty line). Although most people would prefer to use OB drugs, these drugs are not affordable for a large proportion of the population. For instance, 12.3% of the population were found to be unable to afford Lipitor and 10.6% Glucophage. Treatment defects due to high drug costs make disease treatment very costly [39]. A noticeable point this study makes is that withdrawal from treatment, or a reduction of use, is likely among poor households, because of their high sensitivity to price changes [40]. Poverty leads to poor nutrition and unsanitary conditions, and these factors in turn create favorable condition for disease outbreak. In fact, these conditions increase the severity of health problems, such that these two variables intensify each other [41]. This issue increases the likelihood of diseases occurring in several family members or in an individual member of a household. On the other hand, longer and more frequent periods of illness enhance the probability of exposure to catastrophic health expenditure [42]. The present study examined the effect of the age, education and gender of the head of household on the probability of becoming poor due to drug costs. The results showed that female-headed households were almost three times more likely to become impoverished than male-headed households. Typically, the incomes of working women are lower than those of working men [43], which could explain this relationship. With increasing age of the head of household, the probability of becoming poor due to healthcare costs also increased. Furthermore, literate-headed households were less likely to fall into poverty than households with illiterate heads. These two factors have also been evaluated by Hanjani et al., whose results showed that the likelihood of exposure to high healthcare costs fell from 7.8 to 7.3% in literate households. Also, increasing age of the head of household enhances the risk of impoverishment. If the head of household is over 66 years old, the risk of impoverishment increases to 12.2% [31].

In addition, this study found that smaller-sized households were more likely to become impoverished than households with a population of five or more. A greater number of household members usually means a greater number of employed people and therefore increased income. The protective effect of more populated households on impoverishment due to health costs has also been confirmed in a study by Li et al. [44]. However, Ghiasvand’s study showed a direct positive relationship between household size and healthcare costs [43].

Poverty caused by the cost of medication, under different scenarios, was found to be greater in rural areas than urban. This could be due to lower incomes in rural areas. Yazdi-Feizabadi and Akbari also showed that households situated in rural areas were at a greater risk of poverty due to healthcare costs than urban households [11].

## Conclusion

This study showed that some households could not afford the medical costs of drugs and fell below the poverty line due to those costs. Various characteristics of the households had an impact on their impoverishment risk. However, the important point to make is that household characteristics should not affect whether households fall into poverty. The financial protection system against the costs of illness must act in such a way as to enable households to pay for the medications they need regardless of their ability to pay or the type of medication they use. The results of this analysis will be helpful for identifying vulnerable households, to which more attention should be paid.

## Data Availability

The data sets used in this study are freely available from Statistical center of Iran. Data of family costs and income is accessible on the following address: https://www.amar.org.ir/%D8%AF%D8%A7%D8%AF%D9%87%D9%87%D8%A7-%D9%88-%D8%A7%D8%B7%D9%84%D8%A7%D8%B9%D8%A7%D8%AA-%D8%A2%D9%85%D8%A7%D8%B1%DB%8C.

## Abbreviations

LPG: Lowest-priced generic
OB: Original brand
OOP: Out-of-pocket
PPP: Purchasing power parity
